# 838. A Multidisciplinary Approach to the Management of Renal Disease in Patients with HIV

**DOI:** 10.1093/ofid/ofab466.1034

**Published:** 2021-12-04

**Authors:** Yussef Bennani, Annalisa Perez, Hira Hasan, Lauren Richey

**Affiliations:** 1 LSU Health Sciences Center - New Orleans, New Orleans, Louisiana; 2 LSU Health Sciences Center New Orleans, New Orleans, Louisiana; 3 Louisiana State University Health Sciences Center New Orleans, New Orleans, Louisiana; 4 Louisiana State University Health Sciences Center, New Orleans, Louisiana

## Abstract

**Background:**

Chronic kidney disease (CKD) remains an important complication of HIV infection, with up to 30% of people with HIV (PWH) having abnormal renal function. Those with HIV and CKD are reported to have higher mortality than those with either alone. As survival of PWH continues to improve with antiretroviral therapy, additional risk factors for CKD become more prevalent with advancing age. Optimizing management of renal disease in this population to reduce mortality and progression to ESRD has become a more pressing need. Our study describes the implementation of a multidisciplinary HIV/nephrology clinic nested within a Ryan White HIV/AIDS Program-funded clinic on the progression of renal disease in PWH in New Orleans.

**Methods:**

Clinic patients with HIV with at least CKD stage 3 (excluding those with end-stage renal disease) or significant proteinuria were eligible to be referred. Both an HIV primary care provider and a nephrologist evaluated patients at their initial visit, and the subspecialists jointly developed treatment plans. Patients included in the initial analysis were evaluated between January and May 2021. Baseline renal function and proteinuria were obtained, as well as additional studies as appropriate.

**Results:**

A total of 1,968 patients were seen in the HIV clinic during the 18 months prior to the referral period. 305 (15.5%) had an ICD-10 diagnosis code for either CKD or proteinuria. During January – May 2021, 15 patients were referred and 13 evaluated in the multidisciplinary clinic, including 10 men and 3 women. Patients were seen an average of 1.3 times during this time. 8 patients were African-American, and 4 where white. Median age was 59. Median creatinine clearance at baseline was 45 mL/minute. Among those with proteinuria, median proteinuria was 891 mg/g. 8 patients had a diagnosis of hypertension, while 2 had diabetes mellitus. Initial data show a mean improvement in creatinine clearance of 4.2 mL/minute over a mean of 105 days of observation in those with repeat measurements.

**Conclusion:**

There continues to be a high prevalence of CKD in PWH in the era of ART. Given the natural history of kidney disease, an improvement in creatinine clearance is promising. Aggressive co-management of HIV and CKD using a multidisciplinary model may limit progression of CKD and mortality.

**Disclosures:**

**Yussef Bennani, MD, MPH**, **Gilead Sciences** (Scientific Research Study Investigator)**ViiV Healthcare** (Scientific Research Study Investigator)

